# Forecasting new product diffusion using both patent citation and web search traffic

**DOI:** 10.1371/journal.pone.0194723

**Published:** 2018-04-09

**Authors:** Won Sang Lee, Hyo Shin Choi, So Young Sohn

**Affiliations:** Department of Information & Industrial Engineering, Yonsei University, Seoul, Republic of Korea; University of Rijeka, CROATIA

## Abstract

Accurate demand forecasting for new technology products is a key factor in the success of a business. We propose a way to forecasting a new product’s diffusion through technology diffusion and interest diffusion. Technology diffusion and interest diffusion are measured by the volume of patent citations and web search traffic, respectively. We apply the proposed method to forecast the sales of hybrid cars and industrial robots in the US market. The results show that that technology diffusion, as represented by patent citations, can explain long-term sales for hybrid cars and industrial robots. On the other hand, interest diffusion, as represented by web search traffic, can help to improve the predictability of market sales of hybrid cars in the short-term. However, interest diffusion is difficult to explain the sales of industrial robots due to the different market characteristics. Finding indicates our proposed model can relatively well explain the diffusion of consumer goods.

## Introduction

New product demand forecasting is indispensable for production and inventory management. In particular, forecasting the demand for a new product with innovative technology needs to be handled within the perspective of B2B or B2C depending upon the characteristics of the new product. Patents and web search traffic can represent the potential diffusion of technology and consumer demands for innovative technology.

Patents are widely recognized as a quantitative performance measure for technological innovation [1–4]. Among various aspects of patents, the number of patent citations is considered an indicator for a technology diffusion because the more frequently a particular patent is cited by following patents, the more its technology is diffused [5]. As a measure of technology diffusion, it can be used to forecast the sales of a product (for example, mobile phones in South Korea [6] and advanced ceramic powders [7] through the application of a modified Bass model. There is a significant time delay between patent citation and product sales, which makes long-term demand forecasting possible. Thus, it facilitates early strategic decisions of activity management.

In addition, web search traffic can contribute to understanding the diffusion of innovative technologies. Consumers become aware of a new product through various marketing channels, such as advertisement, promotion, social network services (SNS), and blogs. They search words related to a new product using a search engine to obtain detailed and objective information. Thus, for forecasting sales in the near future, in the era of big data, web search traffic is recognized as a new indicator of the degree of consumer recognition and interest, and its effectiveness has been demonstrated in related studies [1, 8–10]. Therefore, in this study the web search traffic was considered a measure of interest diffusion.

However, web search traffic has not been sufficiently applied to the diffusion model, although it can represent the demand for new products via diffusion of interest. Therefore, in our model we apply both web search traffic and patent citation data to increase its ability to predict product sales. This paper proposes a forecasting model for new innovative product diffusion based on both technology diffusion and interest diffusion. The technology diffusion is defined by the number of patent citation, and the interest diffusion is defined by web search traffic. We apply the proposed model to the prediction of sales of representative products containing new technology, namely hybrid cars and industrial robots, in the US market. These items represent high-tech products in the areas of B2C and B2B, respectively. Our study can contribute by improving the prediction performance of diffusion models for new products, which facilitates major strategic decisions. The paper is organized as follows. Section 2 discusses some related work, and Section 3 presents the model specifications used in this research. In Section 4, we explain our data. Section 5 provides the empirical results. Finally, Section 6 concludes with a discussion.

## Literature review

### Technology diffusion and patents citation

Many attempts have been made to measure technology diffusion in industry. However, it is difficult to accurately measure or observe the diffusion effect. Innovators’ decisions are driven by their desire to experience new technologies, while imitators are influenced mostly by the behavior of the people around them. In the content of a patent, the “prior art” is an important element, in that it clearly illustrates the development trace of the invention technology. From a different aspect, the more frequently the patent is cited, the wider the area where the technology spreads is. This implies that the patent has many applications and its value is high [5, 11].

For that reason, patent citation is closely related to diffusion of technology, as shown by analyses in previous studies [12–13]. Jaffe et al. [11] found that inventors learned about cited invention either before or during the development of their invention. This showed that patent citations can be used as a proxy of technological knowledge flows [14]. MacGarvie [15] measured international diffusion of technological knowledge using patent citations. Chang et al. [5] proposed a method to find basic patents related to business methods affecting technology diffusion considering the patent citations using a multi-level citation network. They regarded the citation as tracing the technology spread.

In particular, studies of Lee et al. [6] and Cheng [7] showed that patent citation data can be the leading indicator of the diffusion of a new product or new material, respectively. Lee et al. [6] analyzed the relationship between new product diffusion and diffusion of technology. They used the number of patent citation of code division multiple access (CDMA) technology and the market sales of mobile phones in South Korea for their analysis. Cheng [7] applied the Bass diffusion model using the patent citation data to explore the relationship between new material diffusion of advanced ceramic powders and technology diffusion. Both studies found that the technology diffusion through patent citation can increase the explanation power of the analysis when adopting the Bass diffusion model, which is a well-known diffusion model, is used. As reviewed, the patent citation has been widely utilized to indicate the technology flow and diffusion. Therefore, we regard the patent citation as our indicator for technology diffusion, though it can be limited to completely measure the technology diffusion with the patent citation.

### Customer’s information search behavior and web search traffic

The study of consumer behavior is represented to the methods used by individuals, groups, and organizations to select, purchase, use, and process products, services, ideas, or experiences for the purpose of satisfying their primary and secondary needs [16]. This consumer behavior is affected by cultural, personal, and social factors, and many types of consumer behavior models have been suggested for the purpose of better understanding consumer behavior.

When the purchase decision-making process is examined based on the stage model out of many models that described consumer behavior, it can be broadly divided into five stages: (1) problem awareness, (2) information search, (3) evaluation of alternatives, (4) decision to purchase, and (5) actions following purchase [17].

From the viewpoint of web search traffic, the information search stage in the purchase decision-making process is noted. In this stage, the consumer takes action to gain knowledge after the problem awareness stage, in which the consumer is motivated to act by receiving the marketing stimuli, such as sales promotion campaign or a new product announcement. In case of information search of product applied new technology, the knowledge of product is obtained by advertisement or by directly seeing the product in the initial stage which is not widely spread about knowledge of product. On the other hand, when the knowledge of them is emerged among consumers, it is acquired though the opinions of others, and consumers explore to find objective information. They are important sources in this stage.

With the development of the Internet, consumers began to seek information to enable their efficient and rational decision-making by using a web search. Obtaining information through a web search is extremely efficient in terms of time and money. Web search traffic constitutes the number of searches performed by users, and in the era of big data, it is recognized as a new indicator of the degree of consumer interest. That is, the consumers’ perception degree can be captured through interactive media (i.e., Internet and mobile phones). It is significantly related to the sales of a product.

Choi and Varian [9] described search traffic as highly correlated with a variety of economic indicators, such as automobile sales and international visitor statistics. It can be utilized for forecasting the values of economic indicators in the near future. Jun et al. [10] suggested that the search traffic of a product brand is a more significant factor than patents and news for explaining its sales volume, when using multiple time series regression analysis. Search traffic data can show consumers’ behaviors, as well as their hidden intention. The time series model cannot be easily applied to predict the demand for a new product, because it relies heavily on historical data. However, the diffusion model is effective as a model for predicting the demand of new technology products, even when relatively few data are available.

## Methodology

Rogers [18] defined diffusion as “the process by which an innovation is communicated through certain channels over time among the members of a social system”. Bass [19] proposed an econometric model to explain the innovative product diffusion process. The model can forecast how many customers will finally adopt the new product and when they will adopt it [20]. The model has been applied in a wide variety of industries. It has been shown to capture the diffusion processes of goods and to forecast accurately the product life cycle and sales [21–23].

The Bass model is represented by

$$y(t)=\left\{p+q\frac{Y(t-1)}{M}\right\}\{M-Y(t-1)\}$$ (1)

where y(*t*) denotes the sales of a new product and *Y*(*t* − 1) its cumulative sales, at time *t*. The parameter *M* denotes the market potential, the parameter *p* denotes the innovation coefficient, and *q* denotes the imitation coefficient.

Patent citation is related to the technology diffusion, as examined in studies in the previous literature. Lee et al. [6] defined patent citation as the technology demand and suggested a modified Bass model that uses the number of patent citations.

$$y(t)=\left\lceil p+q\frac{Y(t-1)}{M}+\alpha *patent(t-n)\right\rceil \{M-Y(t-1)\}$$ (2)

where *patent*(*t*) represents the frequency of the related patent citations at time *t* and *n* its time lag. *α* is the effect of the number of patent citations.

Web search traffic represents the spillover of consumer interest in and perception degree of new products. From an increase in web search traffic related to a new product, we can infer that the demand for the new product or consumer intention to purchase it is increasing.

We propose a new diffusion model based on these ideas to predict the growth of a new product’s sales by using the number of patent citations and web search traffic. The proposed extended Bass model is represented by

$$y(t)=\left\lceil p+q\frac{Y(t-1)}{M}+\alpha *patent(t-n)+\beta *traffic(t-m)\right\rceil \{M-Y(t-1)\}$$ (3)

where dependent variable, y(*t*), denotes the sales of a new product at time *t*. While the number of sales is used as a dependent variable in hybrid car case, the sales amount is considered as a dependent variable in industrial robot case as the sales amount can better represent the diffusion patterns of industrial robots than the number of orders. Among independent variables, *Y*(*t* − 1) indicates its cumulative sales at time *t-1*, the parameter *M* denotes the market potential, the parameter *p* denotes the innovation coefficient, and *q* denotes the imitation coefficient. In addition, *patent*(*t*) means the frequency of the related patent citations at time *t* with time lag *n*, and *α* is its effect on the sales of a new product. Also, *traffic*(*t*) represents the frequency of the related web search traffic at time *t* with its time lag *m*, and *β* is the effect of the Web search traffic.

## Data

The selection of appropriate case is important to explain the process of accepting and spreading new technology using patent citation and web search traffic. The analysis target has to be a technology based product which can attract potential interests of customers. This paper considers two new technology-based products with different patterns of interest diffusion, namely hybrid cars and industrial robots. These are representative new-technology products that have been rapidly developing since 2000. However, while hybrid cars are of interest to consumers, industrial robots are only of interest to manufacturers. These products exhibit different web search traffic patterns: searches for hybrid cars experience a gradual rise and fall, whereas those for industrial robots have continuously decreased since 2004. Thus, we consider these cases for our proposed methodology.

Three types of data were collected in this study: patent data, web search traffic data, and sales data. All data were gathered in six-month units, starting from 2004, because web search traffic has only been available from Google since this time. For the hybrid car technology, we selected eight representative frequently cited patents the patents registered at the United States Patents and Trademark Office (USPTO, http://patft.uspto.gov/) as presented in Table 1. We collected the citation frequency of the eight patents from 2004 to 2014. In addition, ten frequently cited patents were chosen from the patents on industrial robots registered at the United States Patents and Trademark Office (USPTO, http://patft.uspto.gov/), as presented in Table 2. We examined the citation frequency of the chosen patents from 2004 to 2014.

**Table 1:** Eight patents related to hybrid car technology.

Number	Title	Application date	Registration date	Citation number
5343970	Hybrid electric vehicle	1992-09-21	1994-09-06	276
4335429	Control apparatus for engine/electric hybrid vehicle	1980-03-12	1982-06-15	213
4351405	Hybrid car with electric and heat engine	1979-11-30	1982-09-28	196
5561380	Fault detection system for electric automobile traction system having floating ground	1995-05-08	1996-10-01	180
5846155	Vehicular drive unit	1996-07-19	1998-12-08	175
5711648	Battery charging and transfer system	1996-11-12	1998-01-27	173
5291960	Hybrid electric vehicle regenerative braking energy recovery system	1992-11-30	1994-03-08	165
5808469	Battery monitor for electric vehicles	1997-03-19	1998-09-15	163

**Table 2:** Top ten patents related to industrial robot technology.

Number	Title	Application date	Registration date	Citation number
7714895	Interactive and shared augmented reality system and method having local and remote access	2003-12-23	2004-09-30	40
6678582	Method and control device for avoiding collisions between cooperating robots	2002-05-30	2004-01-13	36
7979162	Wireless controller and a method for wireless control of a device mounted on a robot	2003-10-07	2006-06-08	33
7040136	Apparatus and a method for calibration of an industrial robot	2002-02-15	2006-05-09	29
7266422	Automated palletizing cases having mixed sizes and shapes	2004-04-09	2007-09-04	29
7236854	Method and a system for programming an industrial robot	2004-01-05	2005-07-07	26
D624104	Industrial robot	2010-03-23	2010-09-21	24
7202442	Cable arrangement for robot arm, and industrial robot utilizing the same	2005-02-22	2007-04-10	20
7967543	Automatic case loader and method for use of same	2008-05-23	2011-06-28	19
8509949	External system for robotic accuracy enhancement	2009-03-23	2013-08-13	19

In addition, we collected monthly Google web search traffic data for “hybrid cars” and “industrial robots” in the United States from 2004–2014 (https://www.google.com/trends). The cases of interest are already predefined by Google, and their monthly web search traffic is provided. The maximum web search traffic for this period was normalized to be 100. In this study, the monthly web search traffic was summed for six-month periods.

Sales data of hybrid cars from 2004–2014 was collected from the U.S. Department of Energy website (http://www.afdc.energy.gov/data/). The increase in the number of hybrid car sales refers to the diffusion of the hybrid car. Sales data for industrial robots from 2004–2014 were collected from the Robotic Industry Association (http://www.robotics.org/). The increase in the number of industrial robots ordered signifies the diffusion of this product.

## Empirical results

Prior to fitting Bass model with data, we respectively examined the correlation between variables for each case. In case of hybrid cars, the correlation between DV and interest diffusion is −0.27, and the correlation between DV and technology diffusion is −0.16. In addition, the correlation between interest diffusion and technology diffusion is −0.18, which is not much higher than the correlation between DV and technology diffusion.

For industrial robots, the correlation between DV and technology diffusion is 0.80, and the correlation between DV and interest diffusion is −0.39, which represents that the modified Bass model can be well applied. On the other hand the correlation between technology diffusion and interest diffusion is −0.45. That correlation is slightly high, and it is necessary to consider this issue when interpreting the result of modified Bass with web searches.

Then, we fitted a Bass diffusion model for each case. The sales number for the hybrid cars and the cost of industrial robots were estimated by a nonlinear least-squares (NLS) estimator based on the proposed model. Table 3 presents the estimation results given by the diffusion model for hybrid cars and industrial robots. In the case of hybrid cars, only the imitation coefficient appeared to be statistically significant. The innovation estimate is 0.00467 and the imitation estimate is 0.06391 for hybrid cars. For the case of industrial robots, the innovation estimate is 0.08014 and the imitation estimate is 0. Both models also show similar levels of MAPEs.

**Table 3:** Estimation results of Bass model.

Hybrid Cars		Industrial Robots	
Parameter	Coefficient	Parameter	Coefficient
*M*	17,354,671	*M*	4,857,909,332
*p*	0.00467	*p*	0.08014
*q*	0.06391 .	*q*	0.44788
MAPE	0.25604	MAPE	0.24283

. : Statistically significant at 10%

For both hybrid cars and industrial robots, the same initial value of *M*, *p*, and *q* was respectively maintained for the estimation. With regard to time lags, the modified Bass model and the modified Bass model with web searches were compared over the same ranges. One time lag amounts to six months. In the case of patent citations, the time lag ranged from 1–16, whereas for web searches, the time lag ranged from 1–8. By comparing the results from different combinations of time lags, we identified the time lag that minimized MAPE value and estimated as many coefficients as they are significant for *M*, *p*, and *q*

Next, we considered model 2, which takes patent citation into account in the Bass model. The sales of hybrid cars and industrial robots were estimated with time lag values after patent citation of *n* = 1, …, 16. In the case of industrial robots, NLS also estimated the parameters for *n* = 1–16. Findings show that MAPE becomes the lowest when the time lag is 9 for hybrid cars, and 13 for industrial robots. Table 4 shows the result of modified Bass model using the patent citation for each case. The entire set of results for the extended Bass model is provided in S1 and S2 Tables.

**Table 4:** Estimation results of the modified Bass model using patent citations.

Hybrid Cars		Industrial Robots	
Parameter	Coefficient	Parameter	Coefficient
*M*	7,679,872 **	*M*	3,968,331,948 *
*p*	0.00807 **	*p*	0.09747 **
*q*	0.13667 **	*q*	0.37543
α	-0.00023	α	0.00399
MAPE	0.18922	MAPE	0.16944

**, *: Statistically significant at 1%, 5%, respectively.

Our findings indicate that the coefficient *α* is not statistically significant for the time lag 9 in hybrid cars. The industrial robots case indicated that only innovation coefficient is significant. The modified Bass model provided better result for industrial robot than hybrid car in terms of the reduction of MAPE.

In the following section, another factor, web search traffic, is added to the model in order to better explain the diffusions of hybrid cars and industrial robots. To take into consideration the spillover effect of the patents and web search traffic, we applied Eq (3) to hybrid cars and industrial robots. We considered several time lag values with *n =* 1, …, 16, because patent citations are relatively suitable for medium- and long-term forecasting. However, we only considered time lag values of *m* = 1, …, 8, as web search traffic is more appropriate for short- and medium-term forecasting. As we took multiple periods into account, the entire results for both cases are provided in S3 and S4 Tables. When *n* = 16 and *m* = 2 were used for hybrid cars, as shown in Eq (4), the coefficients *p*, *q*, *α*, and *β* are statistically significant and positive, as presented in Table 5.

$$y(t)=\left\lceil p+q\frac{Y(t-1)}{M}+\alpha *patent(t-16)+\beta *traffic(t-3)\right\rceil \{M-Y(t-1)\}$$ (4)

**Table 5:** Estimation results of modified Bass model using patent citations and web search traffic.

Hybrid Cars		Industrial Robots	
Parameter	Coefficient	Parameter	Coefficient
*M*	5,494,258 ***	*M*	3,440,460,537 ***
*p*	0.00585 *	*p*	0.12522 ***
*q*	0.07646 ***	*q*	0.51502 *
*α*	0.00063 *	*α*	0.00471 *
*β*	0.00004 ***	*β*	-0.00214
MAPE	0.10575	MAPE	0.14770

***, *: Statistically significant at 0.1%, 5% respectively.

The findings over all time lags show that MAPE is lowest for hybrid cars (*n* = 16 and *m* = 2). However, several time lags other than *n* = 16 and *m* = 2 suggest that not all coefficients are statistically significant. This finding indicates that web search traffic regarding hybrid cars positively affects the diffusion of sales, with a 1 year time lag. Our findings also show that diffusion of technology is associated with the sales of hybrid cars with an 8-year time lag.

**Fig 1:**
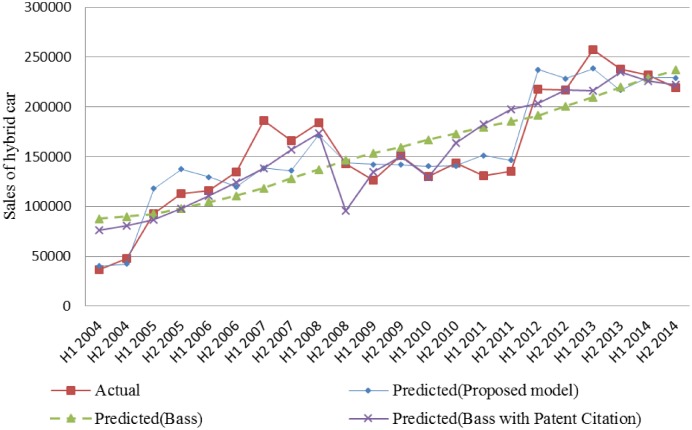
Comparison of actual and predicted sales of hybrid cars.

Fig 1 shows the estimated sales of hybrid cars by Bass model, Bass model with patent citation, and our proposed model against the actual sales. Our proposed Bass model shows a better performance than those of Bass model and Bass model with patent citations.

One can infer that the patent citation and web search traffic can be the leading index with an 8-year time lag and 1-year time lag respectively. The time lag associated with web search traffic is relatively shorter than the time lag associated with the patent citation, and this finding indicates that the web search traffic can be exploited for predicting product sales.

Particularly, after consumers recognize the hybrid car through advertising or word-of-mouth, they search the information on hybrid cars on the Internet and purchase one. In addition, web search could have occurred by potential customers who were curious about the support policy for the hybrid car.

In Eq (5), we consider the spillover effect of the patents and web search traffic for industrial robots on its sales diffusion. Time lag values of *n =* 1–16 and *m* = 1–8 are considered. Multiple periods are examined, and NLS estimated parameters. The entire set of results is given in the appendix.

$$y(t)=\left\lceil p+q\frac{Y(t-1)}{M}+\alpha *patent(t-13)+\beta *traffic(t-2)\right\rceil \{M-Y(t-2)\}$$ (5)

When *n* = 13 and *m* = 4 are used for industrial robots, as shown in Eq (5), the coefficients *p*, *q*, and *α* are statistically significant and positive, as presented in Table 5. However, the coefficient *β* is not statistically significant. With time lags of *n* = 13 and *m* = 4, MAPE is lower than those of other time periods. This indicates that the technology diffusion of industrial robots affects the diffusion of sales, with a 6.5-year time lag. For the industrial robots, the patent citation has shorter time lag than that of hybrid car case. On the other hand, the web search traffic shows more delayed time lags for the product diffusion in industrial robots than that of hybrid cars.

**Fig 2:**
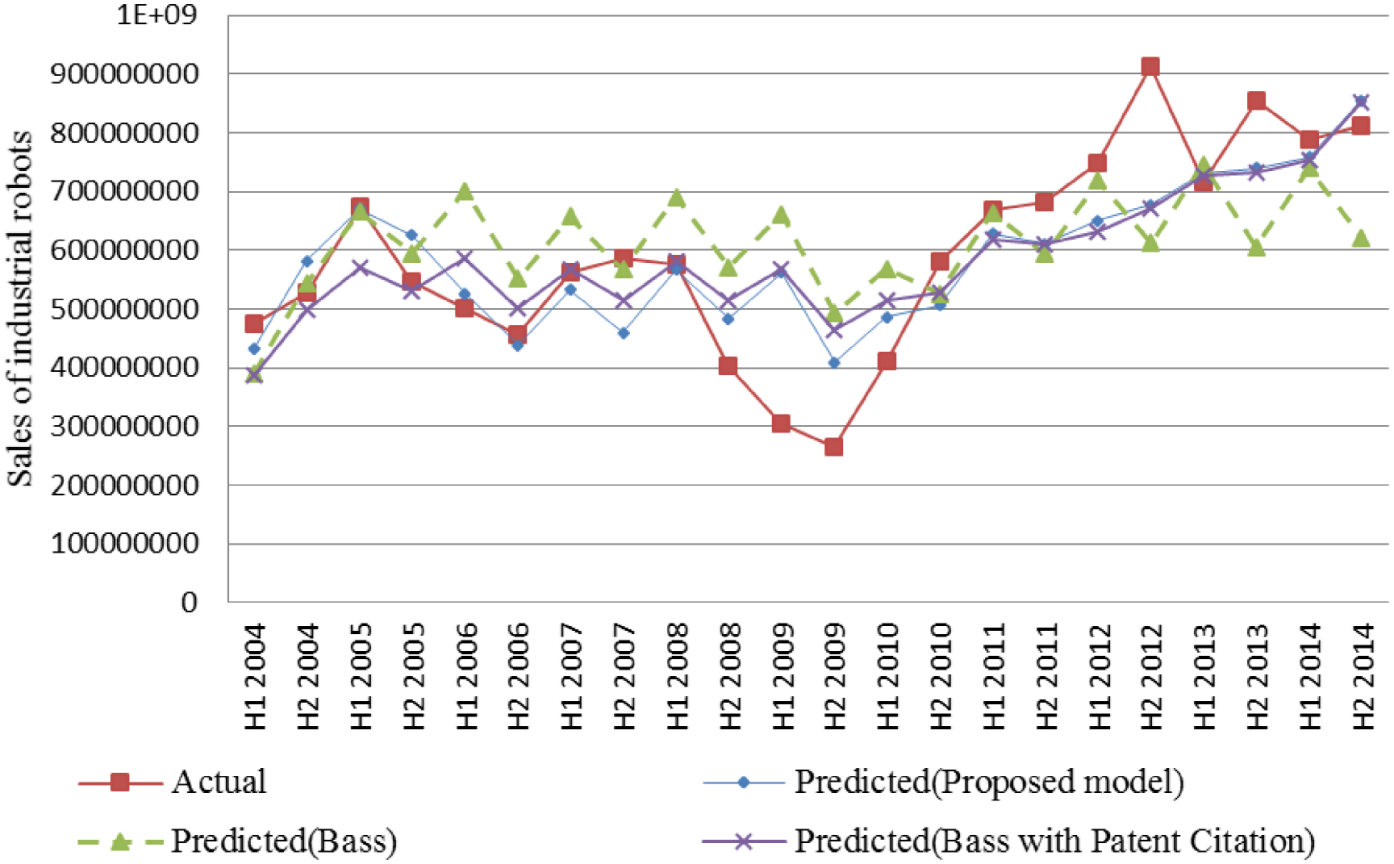
Comparison of actual and estimated sales of industrial robots.

Fig 2 shows both the actual and estimated sales of industrial robots based on the Bass model, Bass model with patent citation, and our proposed model, respectively. Our proposed model with patent citation and web search show better performances than the other models for industrial robots.

Findings can infer that the industrial robots show the shorter time lag for the association with the spread of patent than that of hybrid cars while the hybrid cars have the shorter time lag for the web search traffic than that of industrial robots. Industrial robots, as representative producer goods, are generally used for manufacturing equipment, and purchasers might thoroughly consider the technological characteristics of the robots. It seems that the web search traffic is difficult to be promptly associated with manufacturers heavily. The interest implied by web search traffic is not directly translated into sales in the industrial robots. In particular, as shown in Table 5, the industrial robot case was associated with significant coefficients, p, q, and α, except for the coefficient of web search traffic.

**Fig 3:**
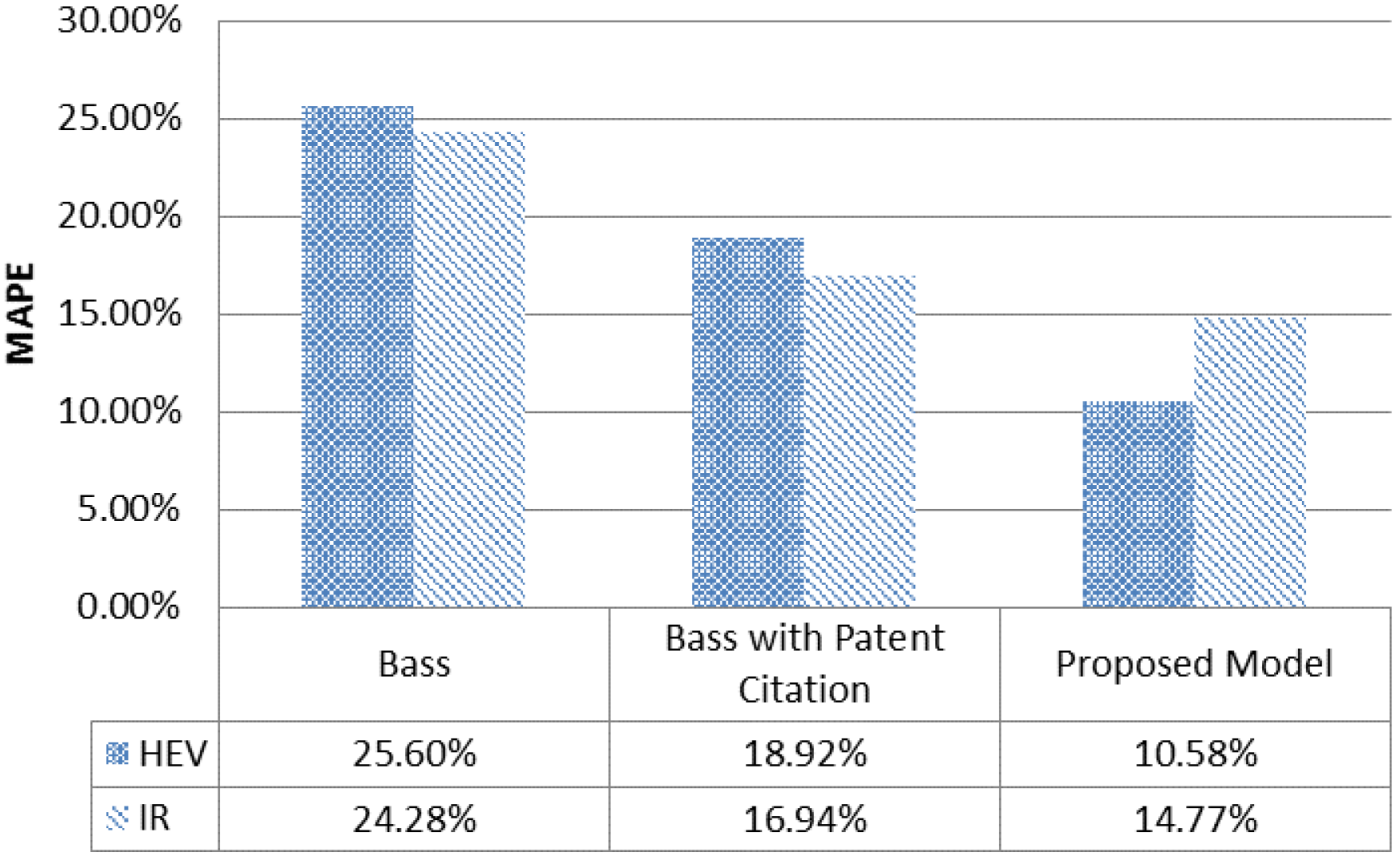
Accuracy comparison for hybrid cars and industrial robots.

In the following figure, we compared the MAPE of three models for each case in order to examine the improvement of model accuracy. Fig 3 shows that Bass model is improved with patent citation and web search traffic in both cases.

For the hybrid cars, MAPEs are respectively 25.60%, 18.92%, and 10.58% for Bass model, Bass model with patent citation, and proposed Bass model. For the industrial robots, MAPEs are 24.28%, 16.94%, and 14.77% for Bass model, Bass model with patent citation, and our proposed model. MAPEs are similar for Bass model and Bass model with patent citation in both cases. Finding shows that our proposed model can finally decrease MAPE for hybrid cars case.

## Conclusion

In this paper, a diffusion model of a new product was proposed that uses web search traffic, focusing on the phenomenon that the consumers’ perception degree of a new product is diffused by various media, as well as patent citations, the number of which represents technology diffusion. In our study, we examined the ability of our proposed diffusion model to predict product sales, by comparing its performance with that of the existing Bass diffusion model. We analyzed the case of both hybrid cars and industrial robots, which are the representative cases of technology oriented products for consumer and manufacturer sides. In particular, an increase in the amount of web search traffic for a specific consumer product can be indirectly interpreted as its potential strength for market success. Interestingly, web search traffic data can be collected or obtained with ease and at little cost. Thus, they can be used to identify the trends or patterns in consumer interests and to forecast new product sales.

Our findings reveal differences between hybrid cars and industrial robots. From our findings, the case of industrial robots has the higher innovation coefficient and imitation coefficient than those of hybrid cars. On the other hand, while all parameters are statistically significant with respect to hybrid cars, but the parameter of web search traffic is not statistically significant with respect to industrial robots. This result corresponds to the finding that web search traffic is important for the sales of hybrid cars, which is the high-tech based consumer goods.

The prediction performance of the proposed model with patent citation and web search traffic is better than those of both Bass model and Bass model with patent citation for both cases. However, both cases differently indicate the reduced MAPE in our proposed model, and it could be due to the different market characteristics.

Our attempt to use interest diffusion is meaningful for new technology-enabled consumer goods, as it shows that the volume of web search traffic can be considered as a measure of consumer interest in the diffusion model. Consumers are interested in the new technology-enabled products, but they can limitedly access the technological interpretation and information in detail. Consumers can rely on the web search for satisfying their information needs. However, for producer goods, web search traffic turned out not to affect the diffusion of industrial robots. This may be because manufacturers seek to satisfy their information needs on the new technology-enabled products with many channels, such as suppliers and product vendors, except for the web search.

The results of this research and their implications are, however, derived from well-known but limited cases. There is a limited ability to generalize the proposed methodology to other products. Applying and modifying this methodology to other products, i.e., non-technology-oriented products, is a subject for future studies. In addition, interpreting the effect of interest diffusion in the case of industrial robots could be somewhat limited due to the abovementioned correlation issue. Different variable for interest diffusion or different approach might be necessary, and those are left for further studies.

## Supporting information

S1 Table Entire results of extended Bass model using patent citations for hybrid cars. (DOCX)

S2 Table Entire results of extended Bass model using patent citations for industrial robots. (DOCX)

S3 Table Entire results of extended Bass model using patent citations and web search traffic for hybrid cars. (DOCX)

S4 Table Entire results of extended Bass model using the patent citation and web search traffic for industrial robots. (DOCX)
