# Supplementary material for: Forecasting new product diffusion using both patent citation and web search traffic
Source: PLoS One. 2018 Apr 9;13(4):e0194723. doi: 10.1371/journal.pone.0194723 (PMC5890978; doi:10.1371/journal.pone.0194723)
Supplement: S1 Table — (DOCX) [file pone.0194723.s001.docx]

**S1 Table. Entire results of extended Bass model using patent citations for hybrid cars**

| Time lag | M | p | q | α | MAPE |
| --- | --- | --- | --- | --- | --- |
| 1 | 17188969 | 0.004687 | 0.064038 | 0.000001 | 0.25563 |
| 2 | 15687061 | 0.004378 | 0.063476 | 0.000022 | 0.23469 |
| 3 | 19154534 | 0.003815 | 0.058205 | 0.000018 | 0.23892 |
| 4 | 20449986 | 0.003825 | 0.058033 | 0.00001 | 0.25109 |
| 5 | 19521225 | 0.004135 | 0.059927 | 0.000006 | 0.25677 |
| 6 | 13382247 | 0.005991 | 0.075178 | -0.00002 | 0.25139 |
| 7 | 8084883 | 0.0088 * | 0.118502 * | -0.000152 | 0.22852 |
| 8 | 7440906 * | 0.008592 ** | 0.136443 ** | -0.000229 | 0.20239 |
| 9 | 7679872 ** | 0.008068 ** | 0.136674 ** | -0.000234 | 0.18922 |
| 10 | 9146152 * | 0.006543 ** | 0.130732 ** | -0.000219 | 0.19083 |
| 11 | 13569519 | 0.004972 | 0.10101 * | -0.000101 | 0.22187 |
| 12 | 21581179 | 0.003302 | 0.085675 * | -0.000052 | 0.23132 |
| 13 | 50720362 | 0.001518 | 0.067412 | -0.000013 | 0.25098 |
| 14 | 20629813 | 0.003912 | 0.063398 | -0.000006 | 0.25649 |
| 15 | 7598527 | 0.010924 | 0.072157 * | 0.00017 | 0.24116 |
| 16 | 5183168 *** | 0.015827 *** | 0.086137 *** | 0.000686 | 0.21418 |

***, **, *, .: Statistically significant at 0.1%, 1%, 5%, 10%, respectively.
