# Supplementary material for: Forecasting new product diffusion using both patent citation and web search traffic
Source: PLoS One. 2018 Apr 9;13(4):e0194723. doi: 10.1371/journal.pone.0194723 (PMC5890978; doi:10.1371/journal.pone.0194723)
Supplement: S2 Table — (DOCX) [file pone.0194723.s002.docx]

**S2 Table. Entire results of extended Bass model using patent citations for industrial robots**

| Time lag | m | p | q | α | MAPE |
| --- | --- | --- | --- | --- | --- |
| 1 | 4191969259 * | 0.090307 * | 0.363727 | 0.003597 | 0.18408 |
| 2 | 4191969259 * | 0.090307 * | 0.363727 | 0.003597 | 0.18408 |
| 3 | 4168289093 * | 0.09059 ** | 0.368056 | 0.003598 | 0.18472 |
| 4 | 4154478129 * | 0.090998 ** | 0.369597 | 0.003602 | 0.18565 |
| 5 | 4146465001 * | 0.090953 ** | 0.372026 | 0.003608 | 0.185 |
| 6 | 4146465001 * | 0.090953 ** | 0.372026 | 0.003608 | 0.185 |
| 7 | 4146465001 * | 0.090953 ** | 0.372026 | 0.003608 | 0.185 |
| 8 | 4146465001 * | 0.090953 ** | 0.372026 | 0.003608 | 0.185 |
| 9 | 4114853953 * | 0.091137 ** | 0.378776 | 0.003625 | 0.18386 |
| 10 | 4075661048 * | 0.092335 ** | 0.380561 | 0.003692 | 0.18142 |
| 11 | 4075661048 * | 0.092335 ** | 0.380561 | 0.003692 | 0.18142 |
| 12 | 4051844249 * | 0.094751 ** | 0.371872 | 0.00382 | 0.17461 |
| 13 | 3968331948 * | 0.097472 ** | 0.375431 | 0.003986 | 0.16944 |
| 14 | 4024769069 * | 0.098182 ** | 0.365235 | 0.003871 | 0.1724 |
| 15 | 3946767830 * | 0.099123 ** | 0.38104 | 0.003897 | 0.17618 |
| 16 | 3899635773 * | 0.101086 ** | 0.38911 | 0.003848 | 0.18135 |

***, **, *, .: Statistically significant at 0.1%, 1%, 5%, 10%, respectively.
