# Supplementary material for: Forecasting new product diffusion using both patent citation and web search traffic
Source: PLoS One. 2018 Apr 9;13(4):e0194723. doi: 10.1371/journal.pone.0194723 (PMC5890978; doi:10.1371/journal.pone.0194723)
Supplement: S3 Table — (DOCX) [file pone.0194723.s003.docx]

**S3 Table. Entire results of extended Bass model using patent citations and web search traffic for hybrid cars**

| Time  Lag  (Pat) | Time  Lag  (Web) | m | p | q | α | β | MAPE |
| --- | --- | --- | --- | --- | --- | --- | --- |
| 1 | 1 | 49397282 | 0.00061 | 0.06278 | -0.00002 | 0.00001 | 0.14485 |
| 1 | 3 | -2447884 | -0.02479 | 0.00501 | 0.00009 | -0.00007 | 0.17218 |
| 1 | 4 | -4815286 | -0.01387 | 0.01295 | 0.00002 | -0.00004 | 0.2011 |
| 1 | 5 | -9968078 | -0.00609 | 0.01986 | -0.00002 | -0.00002 | 0.20374 |
| 1 | 7 | 116707767 | 0.00069 | 0.04454 | 0 | 0 | 0.2534 |
| 1 | 8 | 15649588 | 0.00513 | 0.067 | 0 | 0 | 0.2547 |
| 2 | 1 | 31530053 | 0.00051 | 0.06336 | -0.00001 | 0.00001 | 0.1753 |
| 2 | 3 | -2318692 | -0.02432 | 0.00406 | 0.00006 | -0.00008 | 0.17379 |
| 2 | 4 | -6193046 | -0.00986 | 0.01623 | -0.00001 | -0.00003 | 0.19442 |
| 2 | 5 | -11992660 | -0.00478 | 0.02086 | -0.00003 | -0.00002 | 0.18711 |
| 2 | 7 | 49025128 | 0.00141 | 0.04483 | 0.00001 | 0 | 0.23228 |
| 2 | 8 | 15136735 | 0.00453 | 0.06475 | 0.00002 | 0 | 0.23429 |
| 3 | 1 | 30162798 | 0.00045 | 0.06213 | 0 | 0.00001 | 0.18113 |
| 3 | 4 | -4444656 | -0.01496 | 0.01274 | 0.00004 | -0.00004 | 0.20038 |
| 3 | 5 | -8765127 | -0.00755 | 0.01797 | -0.00002 | -0.00002 | 0.20889 |
| 3 | 8 | 18188833 | 0.00401 | 0.05958 | 0.00002 | 0 | 0.23844 |
| 4 | 1 | 40574182 | 0.00031 | 0.05759 | 0 | 0.00001 | 0.1826 |
| 4 | 6 | -9627437 | -0.00787 | 0.01924 | -0.00001 | -0.00002 | 0.23773 |
| 4 | 8 | 18576082 | 0.00419 | 0.06039 | 0.00001 | 0 | 0.25023 |
| 5 | 1 | 73404013 | 0.00017 | 0.05251 | 0 | 0 | 0.18078 |
| 5 | 2 | -1135923 | -0.03511 | -0.00189 | 0.00012 | -0.00014 | 0.13257 |
| 5 | 3 | -1600795 | -0.03323 | 0.00067 | 0.00012 | -0.0001 | 0.16771 |
| 5 | 8 | 17337015 | 0.00462 | 0.06301 | 0.00001 | 0 | 0.25563 |
| 6 | 1 | 24862480 | 0.00053 | 0.06562 | 0 | 0.00001 | 0.18026 |
| 6 | 7 | 40539162 | 0.00205 | 0.05524 | -0.00001 | 0 | 0.24526 |
| 6 | 8 | 13013750 | 0.00614 | 0.07636 | -0.00002 | 0 | 0.2512 |
| 7 | 1 | 10838697 | 0.001 | 0.09932 * | -0.00008 | 0.00002 | 0.17186 |
| 7 | 2 | 16959093 | 0.00174 | 0.08088 | -0.00004 | 0.00001 | 0.1323 |
| 7 | 3 | 76840726 | 0.00063 | 0.06259 | -0.00001 | 0 | 0.16129 |
| 7 | 6 | 41559596 | 0.00171 | 0.06665 | -0.00003 | 0.00001 | 0.19884 |
| 7 | 7 | 10913132 | 0.00657 | 0.09911 | -0.00016 | 0.00002 | 0.21115 |
| 7 | 8 | 8581786 | 0.00843 * | 0.11241 | -0.00015 | 0 | 0.22844 |
| 8 | 1 | 9088812 * | 0.00088 | 0.11833 ** | -0.00014 | 0.00003 | 0.15538 |
| 8 | 2 | 11879081 | 0.00221 | 0.10002 * | -0.0001 | 0.00002 | 0.11797 |
| 8 | 3 | 17982564 | 0.00247 | 0.08412 | -0.00006 | 0.00001 | 0.14727 |
| 8 | 4 | 55604767 | 0.00103 | 0.06821 | -0.00002 | 0 | 0.17109 |
| 8 | 5 | 156715856 | 0.00041 | 0.0638 | -0.00001 | 0 | 0.17907 |
| 8 | 6 | 22870218 | 0.00295 | 0.07771 | -0.00006 | 0.00001 | 0.18958 |
| 8 | 7 | 9572015 | 0.00697 | 0.11197 * | -0.00018 | 0.00001 | 0.20066 |
| 8 | 8 | 8394105 | 0.00778 * | 0.12415 * | -0.00024 | 0.00001 | 0.19987 |
| 9 | 1 | 9507890 | 0.00171 | 0.11492 ** | -0.00013 | 0.00002 | 0.14185 |
| 9 | 2 | 12239895 | 0.00248 | 0.09885 * | -0.00009 | 0.00002 | 0.11573 |
| 9 | 3 | 16704940 | 0.00279 | 0.08666 | -0.00007 | 0.00001 | 0.14871 |
| 9 | 4 | 28207229 | 0.00206 | 0.07505 | -0.00004 | 0.00001 | 0.17188 |
| 9 | 5 | 40637873 | 0.00159 | 0.07008 | -0.00003 | 0 | 0.1819 |
| 9 | 6 | 17748195 | 0.00379 | 0.08373 | -0.00007 | 0.00001 | 0.19171 |
| 9 | 7 | 8291463 | 0.00765 * | 0.12739 * | -0.00021 | 0 | 0.18947 |
| 9 | 8 | 7823700 * | 0.00798 ** | 0.13424 ** | -0.00023 | 0 | 0.18956 |
| 10 | 1 | 11312095 | 0.00159 | 0.11112 ** | -0.00012 | 0.00002 | 0.13449 |
| 10 | 2 | 15951069 | 0.00195 | 0.09495 * | -0.00008 | 0.00001 | 0.1126 |
| 10 | 3 | 25450854 | 0.00184 | 0.08338 | -0.00005 | 0.00001 | 0.14721 |
| 10 | 4 | 52622538 | 0.0011 | 0.07446 | -0.00002 | 0 | 0.17268 |
| 10 | 5 | 74572561 | 0.00086 | 0.07176 | -0.00002 | 0 | 0.18244 |
| 10 | 6 | 19947095 | 0.00327 | 0.08854 | -0.00007 | 0 | 0.19199 |
| 10 | 7 | 7909472 * | 0.00714 ** | 0.14836 ** | -0.00028 | -0.00001 | 0.18932 |
| 10 | 8 | 7711700 * | 0.00714 ** | 0.15239 ** | -0.00028 | -0.00001 | 0.18508 |
| 11 | 1 | 19028217 | 0.00084 | 0.08537 * | -0.00004 | 0.00001 | 0.15892 |
| 11 | 2 | 112816777 | 0.00029 | 0.06657 | -0.00001 | 0 | 0.12843 |
| 11 | 7 | 11595175 | 0.00567 | 0.11027 | -0.00013 | 0 | 0.22082 |
| 11 | 8 | 9598862 | 0.00645 | 0.12621 * | -0.00017 | -0.00001 | 0.21313 |
| 12 | 1 | 32964122 | 0.00047 | 0.07607 * | -0.00002 | 0.00001 | 0.16252 |
| 12 | 3 | -4179350 | -0.01268 | 0.02236 | 0.0001 | -0.00004 | 0.16191 |
| 12 | 7 | 19556318 | 0.00362 | 0.08842 | -0.00006 | 0 | 0.23107 |
| 12 | 8 | 12292285 | 0.00538 | 0.10947 * | -0.00011 | -0.00001 | 0.22269 |
| 13 | 1 | 540199253 | 0.00003 | 0.06234 | 0 | 0 | 0.17619 |
| 13 | 2 | -1612263 | -0.02395 | 0.00296 | 0.00007 | -0.00011 | 0.13457 |
| 13 | 3 | -2965520 | -0.01754 | 0.01018 | 0.00004 | -0.00006 | 0.16723 |
| 13 | 4 | -5448481 | -0.01149 | 0.01629 | 0.00001 | -0.00003 | 0.1977 |
| 13 | 5 | -7184076 | -0.00976 | 0.01709 | 0 | -0.00002 | 0.21517 |
| 13 | 6 | -8376668 | -0.00914 | 0.02089 | 0.00001 | -0.00002 | 0.23875 |
| 13 | 8 | 22821462 | 0.00325 | 0.0807 | -0.00004 | 0 | 0.24587 |
| 14 | 1 | -1011964 | -0.0281 | -0.00149 | 0.00006 | -0.00017 | 0.15487 |
| 14 | 2 | -1532652 | -0.02498 | 0.00076 | 0.00003 | -0.00012 | 0.13573 |
| 14 | 3 | -2564809 | -0.02063 | 0.00443 | 0 | -0.00007 | 0.16785 |
| 14 | 4 | -6368240 | -0.01013 | 0.01325 | -0.00002 | -0.00003 | 0.19581 |
| 14 | 5 | -9890118 | -0.00735 | 0.01364 | -0.00003 | -0.00002 | 0.21096 |
| 14 | 7 | 200854386 | 0.00042 | 0.04355 | 0 | 0 | 0.25603 |
| 14 | 8 | 18108814 | 0.00441 | 0.06745 | -0.00001 | 0 | 0.25517 |
| 15 | 1 | 8282510 | 0.00156 | 0.07236 ** | 0.00015 | 0.00003 | 0.17249 |
| 15 | 2 | 9494084 | 0.00341 | 0.05881 * | 0.00014 | 0.00003 | 0.13004 |
| 15 | 3 | 11237760 | 0.00456 | 0.04573 | 0.00012 | 0.00002 | 0.15846 |
| 15 | 4 | 13653325 | 0.00477 | 0.03178 | 0.00011 | 0.00002 | 0.18046 |
| 15 | 5 | 14444515 | 0.0052 | 0.02232 | 0.00011 | 0.00002 | 0.195 |
| 15 | 6 | 12726938 | 0.00651 | 0.02365 | 0.00013 | 0.00002 | 0.21361 |
| 15 | 7 | 9001981 | 0.00959 | 0.0484 | 0.00016 | 0.00001 | 0.23663 |
| 15 | 8 | 7766578 | 0.0108 | 0.06804 | 0.00017 | 0 | 0.24223 |
| 16 | 1 | 5353091 *** | 0.00272 | 0.08733 *** | 0.00063 * | 0.00005 ** | 0.14513 |
| 16 | 2 | 5494258 *** | 0.00586 * | 0.07646 *** | 0.00063 * | 0.00004 *** | 0.10575 |
| 16 | 3 | 5627316 *** | 0.0089 ** | 0.06602 *** | 0.00062 * | 0.00004 ** | 0.13446 |
| 16 | 4 | 5774696 *** | 0.01094 *** | 0.05393 ** | 0.00063 | 0.00004 ** | 0.15416 |
| 16 | 5 | 5850486 *** | 0.01245 *** | 0.04389 * | 0.00064 * | 0.00004 ** | 0.16491 |
| 16 | 6 | 5754914 *** | 0.014 *** | 0.04389 * | 0.00066 | 0.00004 ** | 0.18291 |
| 16 | 7 | 5409878 *** | 0.01565 *** | 0.06451 * | 0.00068 | 0.00002 | 0.20713 |
| 16 | 8 | 5230429 *** | 0.01591 *** | 0.08045 * | 0.00069 | 0.00001 | 0.21513 |

***, **, *, .: Statistically significant at 0.1%, 1%, 5%, 10%, respectively.
