# Supplementary material for: Forecasting new product diffusion using both patent citation and web search traffic
Source: PLoS One. 2018 Apr 9;13(4):e0194723. doi: 10.1371/journal.pone.0194723 (PMC5890978; doi:10.1371/journal.pone.0194723)
Supplement: S4 Table — (DOCX) [file pone.0194723.s004.docx]

**S4 Table. Entire results of extended Bass model using the patent citation and web search traffic for industrial robots**

| Time Lag  (Pat) | Time Lag  (Web) | m | p | q | α | β | MAPE |
| --- | --- | --- | --- | --- | --- | --- | --- |
| 1 | 1 | 3713658251 * | 0.10893 * | 0.44679 | 0.00396 | -0.00008 | 0.18428 |
| 1 | 2 | 3321141822 ** | 0.12037 ** | 0.56308 * | 0.00448 * | -0.00018 | 0.17561 |
| 1 | 3 | 3355300030 ** | 0.1279 *** | 0.55083 * | 0.00457 * | -0.00024 | 0.16625 |
| 1 | 4 | 3496246184 ** | 0.1209 *** | 0.52019 * | 0.0045 * | -0.00022 | 0.16364 |
| 1 | 5 | 3693380682 * | 0.11261 ** | 0.4427 | 0.00436 | -0.00014 | 0.17293 |
| 1 | 6 | 3759891339 * | 0.106 ** | 0.4359 | 0.00431 | -0.00011 | 0.17826 |
| 1 | 7 | 3888841566 * | 0.10392 ** | 0.40867 | 0.00422 | -0.00011 | 0.17741 |
| 1 | 8 | 3774849647 * | 0.10724 ** | 0.43429 | 0.00463 | -0.00017 | 0.16797 |
| 2 | 1 | 3713658251 * | 0.10893 * | 0.44679 | 0.00396 | -0.00008 | 0.18428 |
| 2 | 2 | 3321141822 ** | 0.12037 ** | 0.56308 * | 0.00448 * | -0.00018 | 0.17561 |
| 2 | 3 | 3355300030 ** | 0.1279 *** | 0.55083 * | 0.00457 * | -0.00024 | 0.16625 |
| 2 | 4 | 3496246184 ** | 0.1209 *** | 0.52019 * | 0.0045 * | -0.00022 | 0.16364 |
| 2 | 5 | 3693380682 * | 0.11261 ** | 0.4427 | 0.00436 | -0.00014 | 0.17293 |
| 2 | 6 | 3759891339 * | 0.106 ** | 0.4359 | 0.00431 | -0.00011 | 0.17826 |
| 2 | 7 | 3888841566 * | 0.10392 ** | 0.40867 | 0.00422 | -0.00011 | 0.17741 |
| 2 | 8 | 3774849647 * | 0.10724 ** | 0.43429 | 0.00463 | -0.00017 | 0.16797 |
| 3 | 1 | 3705958801 * | 0.10871 * | 0.44943 | 0.00394 | -0.00007 | 0.18508 |
| 3 | 2 | 3320289431 ** | 0.11997 ** | 0.56429 * | 0.00445 * | -0.00018 | 0.1763 |
| 3 | 3 | 3333885622 ** | 0.12889 *** | 0.5586 * | 0.00459 * | -0.00025 | 0.1659 |
| 3 | 4 | 3476358309 ** | 0.12167 *** | 0.52687 * | 0.00452 * | -0.00023 | 0.16332 |
| 3 | 5 | 3668002336 ** | 0.11344 ** | 0.44889 | 0.00439 | -0.00015 | 0.17384 |
| 3 | 6 | 3734009566 * | 0.10667 ** | 0.44213 | 0.00433 | -0.00012 | 0.17925 |
| 3 | 7 | 3864552211 * | 0.1045 ** | 0.41396 | 0.00424 | -0.00011 | 0.17824 |
| 3 | 8 | 3754122497 * | 0.10771 ** | 0.43952 * | 0.00465 | -0.00018 | 0.16889 |
| 4 | 1 | 3709242125 * | 0.10851 * | 0.44843 | 0.00393 | -0.00007 | 0.18605 |
| 4 | 2 | 3330335182 ** | 0.11957 ** | 0.5607 * | 0.00441 * | -0.00017 | 0.17742 |
| 4 | 3 | 3340796019 ** | 0.12847 *** | 0.55653 * | 0.00455 * | -0.00024 | 0.16667 |
| 4 | 4 | 3452673672 ** | 0.12289 *** | 0.53253 * | 0.00457 * | -0.00024 | 0.16271 |
| 4 | 5 | 3641270637 ** | 0.11471 ** | 0.45352 | 0.00443 | -0.00015 | 0.17305 |
| 4 | 6 | 3708361666 * | 0.10769 ** | 0.44658 | 0.00437 | -0.00012 | 0.17854 |
| 4 | 7 | 3841423996 * | 0.10544 ** | 0.41727 | 0.00427 | -0.00012 | 0.17755 |
| 4 | 8 | 3732605586 ** | 0.1086 ** | 0.44295 * | 0.00469 | -0.00018 | 0.16797 |
| 5 | 1 | 3711138232 * | 0.10804 * | 0.44916 | 0.00393 | -0.00007 | 0.18544 |
| 5 | 2 | 3332653019 ** | 0.11911 ** | 0.56091 * | 0.00441 * | -0.00017 | 0.1769 |
| 5 | 3 | 3338969747 ** | 0.12804 *** | 0.5581 * | 0.00454 * | -0.00024 | 0.16598 |
| 5 | 4 | 3449245187 ** | 0.12249 *** | 0.53441 * | 0.00456 * | -0.00024 | 0.16207 |
| 5 | 5 | 3633137567 ** | 0.11506 ** | 0.45675 * | 0.00445 | -0.00016 | 0.17242 |
| 5 | 6 | 3698438960 * | 0.10796 ** | 0.45025 | 0.0044 | -0.00013 | 0.17789 |
| 5 | 7 | 3833783172 * | 0.10565 ** | 0.4202 | 0.00429 | -0.00012 | 0.17692 |
| 5 | 8 | 3727541537 ** | 0.10871 ** | 0.44583 * | 0.00472 | -0.00018 | 0.16707 |
| 6 | 1 | 3711138232 * | 0.10804 * | 0.44916 | 0.00393 | -0.00007 | 0.18544 |
| 6 | 2 | 3332653019 ** | 0.11911 ** | 0.56091 * | 0.00441 * | -0.00017 | 0.1769 |
| 6 | 3 | 3338969747 ** | 0.12804 *** | 0.5581 * | 0.00454 * | -0.00024 | 0.16598 |
| 6 | 4 | 3449245187 ** | 0.12249 *** | 0.53441 * | 0.00456 * | -0.00024 | 0.16207 |
| 6 | 5 | 3633137567 ** | 0.11506 ** | 0.45675 * | 0.00445 | -0.00016 | 0.17242 |
| 6 | 6 | 3698438960 * | 0.10796 ** | 0.45025 | 0.0044 | -0.00013 | 0.17789 |
| 6 | 7 | 3833783172 * | 0.10565 ** | 0.4202 | 0.00429 | -0.00012 | 0.17692 |
| 6 | 8 | 3727541537 ** | 0.10871 ** | 0.44583 * | 0.00472 | -0.00018 | 0.16707 |
| 7 | 1 | 3711138232 * | 0.10804 * | 0.44916 | 0.00393 | -0.00007 | 0.18544 |
| 7 | 2 | 3332653019 ** | 0.11911 ** | 0.56091 * | 0.00441 * | -0.00017 | 0.1769 |
| 7 | 3 | 3338969747 ** | 0.12804 *** | 0.5581 * | 0.00454 * | -0.00024 | 0.16598 |
| 7 | 4 | 3449245187 ** | 0.12249 *** | 0.53441 * | 0.00456 * | -0.00024 | 0.16207 |
| 7 | 5 | 3633137567 ** | 0.11506 ** | 0.45675 * | 0.00445 | -0.00016 | 0.17242 |
| 7 | 6 | 3698438960 * | 0.10796 ** | 0.45025 | 0.0044 | -0.00013 | 0.17789 |
| 7 | 7 | 3833783172 * | 0.10565 ** | 0.4202 | 0.00429 | -0.00012 | 0.17692 |
| 7 | 8 | 3727541537 ** | 0.10871 ** | 0.44583 * | 0.00472 | -0.00018 | 0.16707 |
| 8 | 1 | 3711138232 * | 0.10804 * | 0.44916 | 0.00393 | -0.00007 | 0.18544 |
| 8 | 2 | 3332653019 ** | 0.11911 ** | 0.56091 * | 0.00441 * | -0.00017 | 0.1769 |
| 8 | 3 | 3338969747 ** | 0.12804 *** | 0.5581 * | 0.00454 * | -0.00024 | 0.16598 |
| 8 | 4 | 3449245187 ** | 0.12249 *** | 0.53441 * | 0.00456 * | -0.00024 | 0.16207 |
| 8 | 5 | 3633137567 ** | 0.11506 ** | 0.45675 * | 0.00445 | -0.00016 | 0.17242 |
| 8 | 6 | 3698438960 * | 0.10796 ** | 0.45025 | 0.0044 | -0.00013 | 0.17789 |
| 8 | 7 | 3833783172 * | 0.10565 ** | 0.4202 | 0.00429 | -0.00012 | 0.17692 |
| 8 | 8 | 3727541537 ** | 0.10871 ** | 0.44583 * | 0.00472 | -0.00018 | 0.16707 |
| 9 | 1 | 3682579272 * | 0.1085 * | 0.45736 | 0.00395 | -0.00007 | 0.1844 |
| 9 | 2 | 3315275773 ** | 0.11923 ** | 0.56878 * | 0.00442 * | -0.00017 | 0.17593 |
| 9 | 3 | 3324907510 ** | 0.12792 *** | 0.56466 * | 0.00455 * | -0.00024 | 0.16486 |
| 9 | 4 | 3434397293 ** | 0.12229 *** | 0.54073 * | 0.00456 * | -0.00024 | 0.16221 |
| 9 | 5 | 3614982066 ** | 0.11473 ** | 0.46327 * | 0.00445 | -0.00015 | 0.17351 |
| 9 | 6 | 3681623021 ** | 0.10763 ** | 0.45617 | 0.00439 | -0.00013 | 0.179 |
| 9 | 7 | 3811258943 * | 0.10531 ** | 0.42687 | 0.00428 | -0.00012 | 0.17876 |
| 9 | 8 | 3706118564 ** | 0.10831 ** | 0.45274 * | 0.00469 | -0.00018 | 0.16937 |
| 10 | 1 | 3660581295 * | 0.10944 * | 0.45809 | 0.00401 | -0.00007 | 0.18207 |
| 10 | 2 | 3309899464 ** | 0.11974 ** | 0.56636 * | 0.00447 * | -0.00017 | 0.17385 |
| 10 | 3 | 3317112553 *** | 0.1285 *** | 0.56337 * | 0.00459 * | -0.00024 | 0.16232 |
| 10 | 4 | 3427148567 ** | 0.12284 *** | 0.53903 * | 0.00459 * | -0.00023 | 0.15961 |
| 10 | 5 | 3604148953 ** | 0.11528 ** | 0.46241 * | 0.00448 | -0.00015 | 0.17069 |
| 10 | 6 | 3673105312 ** | 0.10814 ** | 0.45448 | 0.00441 | -0.00012 | 0.1764 |
| 10 | 7 | 3797966168 * | 0.10585 ** | 0.42605 | 0.0043 | -0.00011 | 0.17626 |
| 10 | 8 | 3701841514 ** | 0.10872 ** | 0.45053 * | 0.00468 | -0.00017 | 0.16712 |
| 11 | 1 | 3660581295 * | 0.10944 * | 0.45809 | 0.00401 | -0.00007 | 0.18207 |
| 11 | 2 | 3309899464 ** | 0.11974 ** | 0.56636 * | 0.00447 * | -0.00017 | 0.17385 |
| 11 | 3 | 3317112553 *** | 0.1285 *** | 0.56337 * | 0.00459 * | -0.00024 | 0.16232 |
| 11 | 4 | 3427148567 ** | 0.12284 *** | 0.53903 * | 0.00459 * | -0.00023 | 0.15961 |
| 11 | 5 | 3604148953 ** | 0.11528 ** | 0.46241 * | 0.00448 | -0.00015 | 0.17069 |
| 11 | 6 | 3673105312 ** | 0.10814 ** | 0.45448 | 0.00441 | -0.00012 | 0.1764 |
| 11 | 7 | 3797966168 * | 0.10585 ** | 0.42605 | 0.0043 | -0.00011 | 0.17626 |
| 11 | 8 | 3701841514 ** | 0.10872 ** | 0.45053 * | 0.00468 | -0.00017 | 0.16712 |
| 12 | 1 | 3690736365 * | 0.11004 * | 0.43955 | 0.0041 | -0.00006 | 0.17536 |
| 12 | 2 | 3368466443 ** | 0.1197 ** | 0.53558 * | 0.00451 * | -0.00015 | 0.16782 |
| 12 | 3 | 3354142911 *** | 0.12924 *** | 0.54101 * | 0.00463 * | -0.00022 | 0.15514 |
| 12 | 4 | 3464664912 ** | 0.12372 *** | 0.51769 * | 0.00461 * | -0.00022 | 0.15269 |
| 12 | 5 | 3634894877 ** | 0.1164 ** | 0.44499 * | 0.00452 | -0.00014 | 0.16389 |
| 12 | 6 | 3708182779 ** | 0.10925 ** | 0.43612 | 0.00443 | -0.00011 | 0.17006 |
| 12 | 7 | 3823702759 * | 0.10714 ** | 0.41038 | 0.00434 | -0.0001 | 0.16971 |
| 12 | 8 | 3746374974 ** | 0.10977 ** | 0.43119 * | 0.00465 | -0.00016 | 0.16088 |
| 13 | 1 | 3637893612 ** | 0.11233 ** | 0.44134 | 0.00426 | -0.00006 | 0.17035 |
| 13 | 2 | 3350239117 ** | 0.12106 ** | 0.5311 * | 0.00463 * | -0.00014 | 0.16334 |
| 13 | 3 | 3331056660 *** | 0.13078 *** | 0.53886 * | 0.00475 * | -0.00022 | 0.14985 |
| 13 | 4 | 3440460537 *** | 0.12522 *** | 0.51502 * | 0.00471 * | -0.00021 | 0.1477 |
| 13 | 5 | 3600440536 ** | 0.11802 ** | 0.44416 * | 0.00462 * | -0.00014 | 0.15827 |
| 13 | 6 | 3675375121 ** | 0.11078 ** | 0.43404 * | 0.00453 | -0.0001 | 0.16491 |
| 13 | 7 | 3779823751 * | 0.10879 ** | 0.40995 | 0.00443 | -0.0001 | 0.16472 |
| 13 | 8 | 3716640712 ** | 0.1112 ** | 0.42874 * | 0.0047 | -0.00015 | 0.15639 |
| 14 | 1 | 3656855985 * | 0.11429 ** | 0.43601 | 0.00417 | -0.00007 | 0.17327 |
| 14 | 2 | 3380631620 ** | 0.1223 ** | 0.5209 | 0.00452 * | -0.00014 | 0.16683 |
| 14 | 3 | 3365329093 *** | 0.13175 *** | 0.52751 * | 0.00462 * | -0.00022 | 0.15256 |
| 14 | 4 | 3487053975 ** | 0.12573 *** | 0.50159 * | 0.00456 * | -0.00021 | 0.15074 |
| 14 | 5 | 3656965326 ** | 0.11822 ** | 0.43086 * | 0.00447 | -0.00013 | 0.16208 |
| 14 | 6 | 3743699564 * | 0.11075 ** | 0.419 | 0.00435 | -0.0001 | 0.16848 |
| 14 | 7 | 3851079263 * | 0.1086 ** | 0.39594 | 0.00426 | -0.00009 | 0.16857 |
| 14 | 8 | 3795114661 * | 0.11085 ** | 0.41262 | 0.00448 | -0.00013 | 0.16108 |
| 15 | 1 | 3555182130 ** | 0.1176 ** | 0.46294 | 0.00422 | -0.00008 | 0.17698 |
| 15 | 2 | 3302237764 ** | 0.12463 ** | 0.54991 * | 0.00457 * | -0.00016 | 0.17039 |
| 15 | 3 | 3312154381 *** | 0.13311 *** | 0.54746 * | 0.00464 * | -0.00023 | 0.15613 |
| 15 | 4 | 3435527616 ** | 0.12667 *** | 0.51916 * | 0.00458 * | -0.00021 | 0.15376 |
| 15 | 5 | 3596033661 ** | 0.11898 ** | 0.44774 * | 0.00448 | -0.00013 | 0.16516 |
| 15 | 6 | 3683085068 ** | 0.11131 ** | 0.43477 | 0.00436 | -0.0001 | 0.17151 |
| 15 | 7 | 3784751195 * | 0.10905 ** | 0.41159 | 0.00426 | -0.00008 | 0.17233 |
| 15 | 8 | 3732342333 ** | 0.11118 ** | 0.42816 * | 0.00448 | -0.00013 | 0.16532 |
| 16 | 1 | 3454832426 ** | 0.12324 ** | 0.48857 | 0.00422 | -0.0001 | 0.18208 |
| 16 | 2 | 3229625677 ** | 0.12873 ** | 0.57584 * | 0.00455 * | -0.00018 | 0.17583 |
| 16 | 3 | 3266561339 *** | 0.13608 *** | 0.56241 * | 0.0046 * | -0.00024 | 0.16121 |
| 16 | 4 | 3400933505 ** | 0.12879 *** | 0.52918 * | 0.00451 * | -0.00022 | 0.15925 |
| 16 | 5 | 3560085553 ** | 0.12072 ** | 0.45612 * | 0.00441 | -0.00013 | 0.17047 |
| 16 | 6 | 3656406367 ** | 0.11259 ** | 0.44043 | 0.00427 | -0.00009 | 0.17677 |
| 16 | 7 | 3756357005 * | 0.11007 ** | 0.41722 | 0.00417 | -0.00008 | 0.17822 |
| 16 | 8 | 3711978664 * | 0.11199 ** | 0.43221 | 0.00434 | -0.00012 | 0.17211 |

***, **, *, .: Statistically significant at 0.1%, 1%, 5%, 10%, respectively.
